# Seeing Beyond Morphology-Standardized Stress MRI to Assess Human Knee Joint Instability

**DOI:** 10.3390/diagnostics11061035

**Published:** 2021-06-04

**Authors:** Eva-Maria Winkelmeyer, Justus Schock, Lena Marie Wollschläger, Philipp Schad, Marc Sebastian Huppertz, Niklas Kotowski, Andreas Prescher, Christiane Kuhl, Daniel Truhn, Sven Nebelung

**Affiliations:** 1 Department of Diagnostic and Interventional Radiology, Aachen University Hospital, 52074 Aachen, Germany; eva.winkelmeyer@rwth-aachen.de (E.-M.W.); pschad@ukaachen.de (P.S.); mhuppertz@ukaachen.de (M.S.H.); niklas.kotowski@rwth-aachen.de (N.K.); ckuhl@ukaachen.de (C.K.); dtruhn@ukaachen.de (D.T.); 2 Department of Diagnostic and Interventional Radiology, Medical Faculty, University Dusseldorf, D-40225 Duesseldorf, Germany; Justus.Schock@med.uni-duesseldorf.de (J.S.); Lena.Wollschlaeger@med.uni-duesseldorf.de (L.M.W.); 3 Institute of Anatomy, RWTH Aachen University, 52074 Aachen, Germany; aprescher@ukaachen.de

**Keywords:** knee joint, anterior cruciate ligament, magnetic resonance imaging, physiology, biomechanical phenomena

## Abstract

While providing the reference imaging modality for joint pathologies, MRI is focused on morphology and static configurations, thereby not fully exploiting the modality’s diagnostic capabilities. This study aimed to assess the diagnostic value of stress MRI combining imaging and loading in differentiating partial versus complete anterior cruciate ligament (ACL)-injury. Ten human cadaveric knee joint specimens were subjected to serial imaging using a 3.0T MRI scanner and a custom-made pressure-controlled loading device. Emulating the anterior-drawer test, joints were imaged before and after arthroscopic partial and complete ACL transection in the unloaded and loaded configurations using morphologic sequences. Following manual segmentations and registration of anatomic landmarks, two 3D vectors were computed between anatomic landmarks and registered coordinates. Loading-induced changes were quantified as vector lengths, angles, and projections on the x-, y-, and z-axis, related to the intact unloaded configuration, and referenced to manual measurements. Vector lengths and projections significantly increased with loading and increasing ACL injury and indicated multidimensional changes. Manual measurements confirmed gradually increasing anterior tibial translation. Beyond imaging of ligament structure and functionality, stress MRI techniques can quantify joint stability to differentiate partial and complete ACL injury and, possibly, compare surgical procedures and monitor treatment outcomes.

## 1. Introduction

Ligament injuries are common and constitute the majority of sports-related knee joint injuries [1,2]. National registries recording the incidence of anterior cruciate ligament (ACL)-injuries have reported mean incidences of 29–38 per 100,000 people [3].

Because of its excellent soft tissue contrast, non-invasiveness, lack of ionizing radiation, and substantial diagnostic capabilities, MRI is the imaging modality of choice in patients with suspected knee and ACL injuries. Its diagnostic performance, however, is variable. While complete ACL tears are identified with excellent sensitivity and specificity [4], partial ACL tears are diagnosed with considerably poorer sensitivity and specificity [5,6], even if standard clinical MRI protocols are supplemented by oblique or 3D volume sequences [7,8]. With an estimated prevalence of 20–47% of all ACL injuries [9], partial ACL tears involve the anteromedial or posterolateral bundle [10] or up to 75% of the ligament’s diameter [11]. Due to their various injury patterns and imaging features, partial ACL tears are oftentimes indistinguishable from complete injuries, mucoid degeneration or even the normal ACL [12]. After reviewing 300 patients, Dejour et al. concluded that it was not possible to identify partial ACL tears (as confirmed arthroscopically) in preoperative MRI scans [13]. However, there is an important clinical need for an accurate distinction of partial from complete ACL injuries as it impacts the type of treatment, i.e., conservative versus surgical, and the exact operative technique and workflow for ACL reconstruction [9,13,14,15]. Consequently, patients with suspected ACL injury may undergo arthroscopy [9] that they could be spared if preoperative diagnostics were improved [10,13,16,17,18].

One potential approach to render joint assessment more functional is to image the joint under loading. Currently, stress radiography is the imaging modality of choice to study anterior tibial translation (ATT) as a surrogate of ACL integrity and joint stability. Quantification of ATT is prone to inaccuracy [19], not least because of test-retest reproducibility error of up to 2.4 mm and, consequently, highly variable intra- and inter-reader variability [20]. With the indication for ACL-reconstructive surgery widely accepted for side-to-side differences of ATT of ≥5 mm (alongside a positive pivot-shift test) [21], this lack of accuracy renders stress radiography unsuitable to meet the clinical needs of precision and standardization. Stress MRI techniques that combine MRI and loading are promising because they help assess both ligament structure and functionality. Earlier prototypical devices using leg splints [16], weighted orthoses [22], and loading fixtures [17,23] were primarily focused on orthopaedic aspects and distinct patient cohorts, comparing patients with complete ACL injuries and healthy individuals [16,17,22]. Despite variable levels of technical sophistication and standardization, these pioneering studies indicated that such approaches could be clinically useful. However, because they excluded partial ACL injury, acquired only single pulse sequences at relatively low resolutions, and because they involved manual measurements by single readers, these studies may not exploit the full potential of stress MRI techniques.

Therefore, this study applied morphologic MRI, combined with pressure-controlled loading and comprehensive image post-processing, on an in-situ model of graded ACL injury to analyse the diagnostic value of stress MRI in differentiating partial and complete ACL injury in a basic research context. We hypothesized that (i) loading induces distinct changes in femorotibial kinematics with increasing grades of ACL injury and that (ii) these changes may be quantified accurately using computed 3D and manual 2D measurements.

## 2. Materials and Methods

### 2.1. Study Design

This study was designed as an ex-vivo experimental study on human cadaveric knee joint specimens and carried out from February 2020–July 2020. Local Institutional Review Board approval (Ethical Committee, RWTH Aachen University, EK180/16, issued on 13 July 2016) and written informed consent by the body donors were available at study initiation. All measurements were performed in accordance with the relevant local guidelines and regulations.

### 2.2. Loading Device and Human Knee Joint Specimens

**Figure 1:**
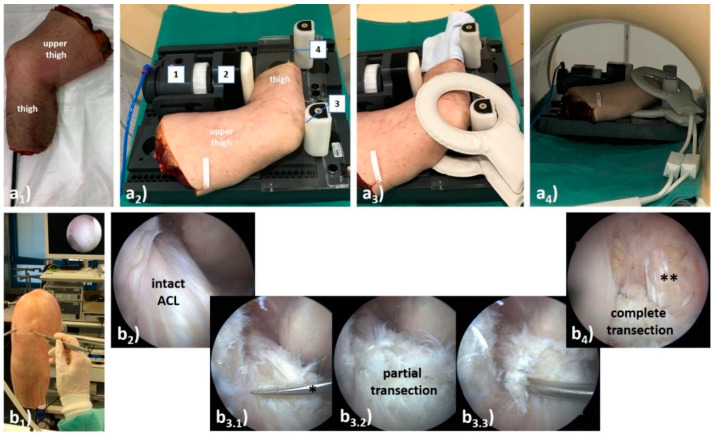
Synopsis of MR imaging setup (**a**) and arthroscopic transection model of the anterior cruciate ligament (ACL) (**b**). (**a**) After extension of the tibia with a polyvinyl-chloride medullary rod (**a_1_**), the unfixed human cadaveric knee joint specimen was positioned on the MRI-compatible, pressure-controlled loading device (**a_2_**) and imaging was performed using dual-coils (**a_3_**). The knee joint was loaded by anterior displacement of the lower thigh relative to the fixed upper thigh. Once attached to the in-house pressure supply, the pneumatics (1) and its padded load applicator (2) were brought in contact with the proximal third of the calf to displace the lower thigh anteriorly relative to the femur. Opposite counter-bearings were positioned at two locations, i.e., at the patella (3) and the extended tibia (above the ankle) (4). Once fully set up and operational, the device was centrally positioned in the MRI scanner’s bore (**a_4_**). (**b)** Following standard portal placement during standard knee arthroscopy (**b_1_**), the ACL was identified and synovectomised if necessary (**b_2_**). In a two-stage procedure, the ACL was subject to partial transection (**b_3_**) followed by complete transection (**b_4_**). During partial transection, approximately 50 % of the ACL was cut using arthroscopic scissors (*) (**b_3.1_**), and the remaining fibres were visualized (**b_3.2_**) and functionally evaluated by arthroscopic probing (**b_3.3_**). After complete transection, the ACL was slack and was flattened in its course so that the PCL was visible in its entirety (**).

Details of the MRI-compatible pressure-controlled loading device have been published before [24]. Set up as a leverage mechanism, the central pneumatics actuate a padded pressure applicator by control of set pressure levels, while two variably positioned opposite counter-bearings serve as fixed points. Ten fresh (i.e., unfixed) and unpaired human knee joint specimens were consecutively obtained via the Institute of Anatomy (RWTH Aachen University, Aachen, Germany) from body donors who had deceased due to unrelated medical conditions. The specimens’ characteristics were 7 women, 3 men, 5 right, and 5 left. Mean age at death was 79.0 ± 13.9 years (range, 49–94 years). Exclusion criteria were signs of previous surgery of the knee, orthopedic implants, and signs suggestive of chronic ACL deficiency as outlined below. For logistical reasons, specimens were frozen at −20 °C for no longer than two weeks prior to the study. Through equilibration at room temperature for at least 12 h, depending on specimen size, specimens were fully thawed before the MRI studies. Tibial diaphyses were extended by tapered polyvinyl-chloride medullary rods driven into the medullary cavity and fixed using liquid polymethyl-methacrylate (Technovit-3040, Heraeus-Kulzer) (Figure 1(a_1_)). Specimens were placed in the lateral position with the padded pressure applicator at the proximal third of the calf, and the counter-bearings at the patella and the extended lower thigh (Figure 1(a_2_)). Joint flexion of approximately 90° was maintained by use of sandbags and positioning aids. Device components were brought in loose contact with the specimen.

Specimen size calculation was performed on the initial three specimens (power 0.8; probability of Type-I-error 0.05; assumed effect size 1.6, two-tailed procedure) and minimum sample size was determined as eight based on a dedicated online software (www.statstodo.com, accessed on 12 September 2020).

### 2.3. MRI Studies

Standard clinical multi-channel knee coils could not be used with the loading device. Therefore, multi-purpose phased array dual-coils (Sense-Flex M, Philips) were placed around the joint (Figure 1(a_3_)). The specimen-loaded device was positioned centrally in a clinical 3.0T scanner (Achieva, Philips) (Figure 1(a_4_)). MRI measurements were performed in the unloaded (δ_0_) and loaded (δ_1_) configurations. Following imaging in the δ_0_-configuration for reference purposes, pressure was set to 3.23 bar, resulting in an effective force of 147 N [=15 kP] [24] that was applied at the proximal calf to displace the tibia anteriorly. After observing an equilibration period of 5 min, imaging in the δ_1_-configuration was performed. Table 1 details the imaging protocol for each joint and configuration: following the acquisition of scout views, proton density-weighted fat-saturated, T1-weighted, and T2-weighted 2D turbo spin-echo sequences were acquired. Signs suggestive of chronic ACL deficiency, i.e., absent, conspicuously hypointense or fragmented ACL morphologies [25], were assessed during the initial MRI study by SN (clinical radiologist, 8 years of experience) and not found to be present. Of note, the sagittal T1-weighted sequence was segmented to generate the specimen-specific 3D joint models used for computation of the 3D measures as detailed below. Measurements were performed at room temperature, which was monitored during one measurement series (20.4 ± 0.7 °C).

MRI measurements were performed sequentially in three ACL conditions, (i) ACL-intact, (ii) partially ACL-deficient and (iii) completely ACL-deficient. In total, each specimen was subject to six MRI measurements, i.e., at the δ_0_- and δ_1_-configurations in each ACL condition. Particular attention was paid to realize similar imaging and loading conditions for the same specimen and between specimens and any residual tibial displacement, if present, was repositioned. Magnet time per specimen, ACL condition, and configuration was approximately 30 min and total magnet time per specimen was 3 h. In-between the measurements, specimens were stored refrigerated at 4 °C and allowed to equilibrate for at least 2 h. The sequential measurement sessions were completed within 48–60 h.

### 2.4. ACL Transection Model

Standard arthroscopy was performed in-between the sequential MRI measurements (Figure 1(b_1_)) by SN (common trunk-trained orthopedic surgeon). Following access to the joint via standard anteromedial and anterolateral portals, the ACL was identified (Figure 1(b_2_)) and synovectomized for full visualization. For partial ACL transection during the first arthroscopy session, the ACL was cut at mid-substance level to approximately 50% of its cross-sectional diameter using arthroscopic straight-tip scissors (Arthrex) (Figure 1(b_3_)). No particular attention was directed at differentiating the ACL bundles. After partial transection, functional integrity of the remaining fibers was assessed by arthroscopic probing. During the second arthroscopy session, the ACL was completely transected (Figure 1(b_4_)). Once the procedure was completed, the joint was thoroughly irrigated, excess fluid removed, and portals sutured.

### 2.5. Image Post-Processing and Analysis

#### 2.5.1. Computed 3D Measures

For each configuration, femur and tibia were manually segmented on sagittal T1-weighted images by EMW (medical student, two years of experience in musculoskeletal imaging) using the semiautomatic segmentation function of ITK-SNAP (v3.8, Cognitica) [26]. Femoral and tibial central bone axes and anatomic landmarks, i.e., the centres of the tibial tuberosity and the tip of the cartilage-covered femoral trochlea, were identified and registered. Inter-measurement and intra-reader deviations of these coordinates and landmarks was low, ranging from 0.73 to 1.67 mm, indicating excellent reproducibility (Supplementary Material Table SF1). Two fixpoints were computed as the intersections of the central bone axes with the articulating femoral and tibial surfaces, i.e., femoral and tibial axis-bone-intersections. Following training on three knee joints in all configurations, EMW performed the segmentations and defined the coordinates and landmarks that were reviewed for accuracy and consistency by SN. No corrections were necessary.

Based on these data, 3D joint models were implemented for each specimen, loading configuration, and ACL condition, to quantify the knee joint’s motional changes as a function of loading and ACL status. 3D Euclidean vectors between the anatomic landmarks (“vector_landmarks”) and between the axis-bone-intersections (“vector_ABI”) were determined and quantified in their respective lengths. In addition, these vectors’ projections onto the Cartesian axes x (“x_landmarks”, “x_ABI”), y (“y_landmarks”, “y_ABI”), and z (“z_landmarks”, “z_ABI”) were quantified to determine the respective vector’s projective length in a defined dimension. Similarly, the projected angles between the central bone axes (“xz-angle”, “yz-angle”, “xy-angle”) were quantified. Here, yz-angles indicate sagittal alignment and knee joint flexion, while (by convention) xz-angles indicate the medial-sided angle, coronal alignment, and, thus, varus or valgus deviation. Correspondingly, xy-angles indicate axial alignment and knee joint rotation.

**Figure 2:**
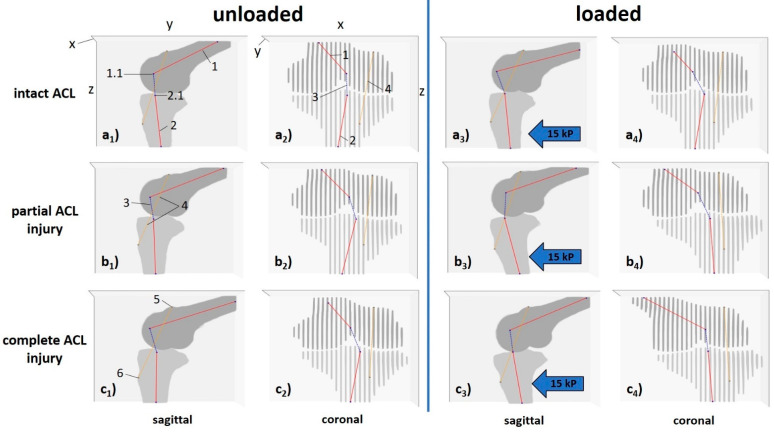
Visualization of 3D measures to quantify knee joint motion as a function of loading and ACL status (left knee joint). In this representative knee joint, unloaded (**a_1_**–**c_1_**,**a_2_**–**c_2_**) and loaded configurations (**a_3_**–**c_3_**,**a_4_**–**c_4_**) are displayed for the ACL-intact (**a**), partially (**b**) and completely ACL-deficient configurations (**c**). Femur and tibia are visualized along the *y*-*z*-plane (corresponding to the sagittal view, **a_1_**–**c_1_**,**a_3_**–**c_3_**,) and the *x*-*z*-plane (corresponding to the coronal view, **a_2_**–**c_2_**,**a_4_**–**c_4_**). Following manual segmentations of the femur (dark grey) and tibia (light grey) and delineation of the central bone axes (red), the femoral and tibial axis-bone-intersections (ABI) were computed (black crosses). Similarly, the centres of the tibial tuberosity and the tip of the cartilage-covered trochlea were manually identified (dark grey crosses). 3D Euclidean vectors connected the femoral and tibial axis-bone-intersections (dashed blue lines) and the anatomic landmarks (dashed orange lines). Blue block arrows indicate the direction of force applied to the proximal calf. Designated axes as given in a_1_ and a_2_ range from 0–30 (*x*-axis) and 0–448 (*y*- and *z*-axes) pixels, respectively. Sliced appearance of femur and tibia in (**a_2_**–**c_3_**,**a_4_**–**c_4_**) is due to interslice gaps after secondary reconstruction. 1—central femoral bone axis, 1.1—femoral axis-bone-intersection, 2—central tibial bone axis, 2.1—tibial axis-bone-intersection, 3—vector_ABI, 4—vector_landmarks, 5—tip of the cartilage-covered trochlea, 6—centre of the tibial tuberosity. Same knee joint specimen as in Figure 3, Figure 4 and Figure 5.

**Figure 3:**
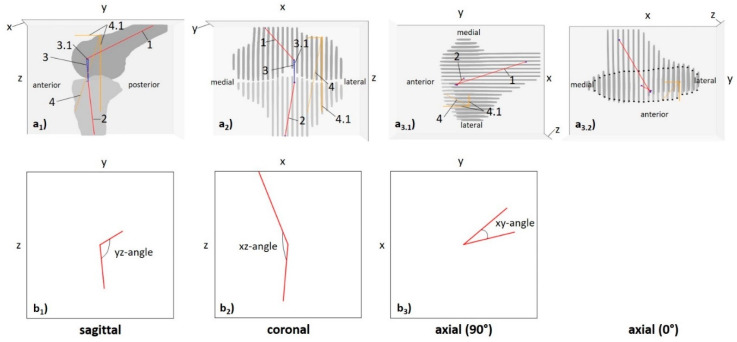
Detailed quantification of 3D measures to quantify knee joint motion (left knee joint). Femur (dark grey) and tibia (light grey) are visualized along the *y*-*z* (**a_1_**), *x*-*z* (**a_2_**), and *x*-*y* planes (**a_3_**). Red lines give central bone axes, black crosses the intersections of central bone axes and bone, grey crosses the anatomic landmarks, i.e., the tip of the cartilage-covered femoral trochlea and the centre of the tibial tuberosity, dashed blue lines the vectors between the axis-bone-intersections, and dashed orange lines the vectors between the anatomic landmarks. Besides vector magnitudes (”length_ABI” [dashed blue lines]; ”length_landmarks” [dashed orange line]), vector projections along the *x*-, *y*-, and *z*-axes were quantified as “x_ABI”, “y_ABI”, “z_ABI” (solid blue lines) or “x_landmarks”, “y_landmarks”, and “z_landmarks” (solid orange lines). Positive values for the measure along the *x*-, *y*-, and *z*-axis indicate a more lateral, posterior, and proximal position of the femoral point as compared to the tibial point. Axial views are visualized in 90° (**a_3.1_**) and 0° rotation (**a_3.2_**). Due to significant overlap and obscured delineation of femur and tibia (a_3.1_), the tibial contours are indicated by black dots (**a_3.2_**). Following their projections onto the *yz*- (**b_1_**, “sagittal”), *xz*- (**b_2_**, “coronal”), and *xy*-planes (**b_3_**, “axial”), angles between the femoral and tibial central bone axes were quantified, as well. In this exemplary knee joint configuration (ACL-intact δ_0_-configuration), the angles were quantified as *yz* = 113°, *xz* = 175°, and *xy* = 41°. For *yz*-angles, large values close to 180° indicate nearly full extension of the knee joint, while lower angles between 180° and 90° indicate increasing knee joint flexion angles and an *yz*-angle of 90° the perpendicular orientation of the central bone axes in the sagittal plane. For *xz*-angles, large values close to 180° indicate nearly parallel orientations of the femoral and tibial central bone axes, while values close to 90° indicate nearly right-angled orientations. By convention, *xz*-angles were defined as the medial-sided angles between the central bone axes in the coronal plane. For *xy*-angles, values close to 0° indicate nearly parallel orientations of the central bone axes in the axial plane, while increasing *xy*-angles indicate increasing external rotation. Of note, as projective measures, these angles are prone to cross-plane effects introduced by coronal, sagittal, and axial alignment. 1—central femoral bone axis, 2—central tibial bone axis, 3—vector_ABI, 3.1—vector projections of vector_ABI, 4—vector_landmarks, 4.1—vector projections of vector_landmarks. Same knee joint specimen as in Figure 2, Figure 4 and Figure 5.

Figure 2 and Figure 3 visualize the segmentation outlines, anatomic landmarks, fixpoints, vectors, and angles for multidimensional evaluation of knee joint motion and their exact quantification in a representative knee joint, while Supplementary Text 1 gives the technical details of the post-processing methodology. Initial voxel-wise measurements were converted to mm based on voxel size of 0.36 × 0.36 × 3.0 mm³ and an inter-slice gap of 0.3 mm.

#### 2.5.2. Manual 2D Reference Measures

**Figure 4:**
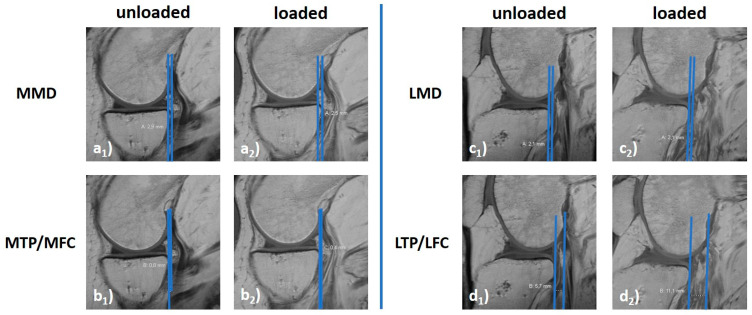
Exemplary manual measures to quantify knee joint motion. For the medial (**a**,**b**) and lateral (**c**,**d**) femorotibial compartments, loading-induced anterior translation of the tibia was quantified relative to the posterior horns of the menisci (MMD [**a**], LMD [**c**]) and the femoral condyles (MTP/MFC [**b**], LTP/LFC [**d**]). Unloaded (**a_1_**–**d_1_**) and loaded configurations (**a_2_**–**d_2_**). MMD and LMD quantify the respective femorotibial compartment’s horizontal distance between the tangential lines at the base of the meniscus’ posterior horn and the posterior margin of the tibial plateau’s articular surface. Positive values indicate a more anterior position of the posterior horn relative to the tibial plateau, while negative values indicate a more posterior position. MTP/MFC and LTP/LFC give the horizontal distance between tangential lines to the posterior margins of the femoral condyle and the tibial plateau’s articular surface. Positive values indicate a more anterior position of the femoral condyle relative to the tibial plateau, while negative values indicate a more posterior position. [mm]. Same knee joint specimen as in Figure 2, Figure 3 and Figure 5 (ACL-intact δ_0_-configuration).

Secondary signs of ACL deficiency were analyzed separately by two readers (EMW and SN). Blinding proved infeasible because the joints’ configuration was readily discernible. Mid-sagittal slices of the medial and lateral femorotibial compartments were identified and used to quantify knee joint motion as before [16]: (i) lateral meniscus displacement distance (LMD); (ii) medial meniscus displacement distance (MMD); (iii) lateral tibial plateau displacement distance relative to the lateral femoral condyle (LTP/LFC); and (iv) medial tibial plateau displacement distance relative to the medial femoral condyle (MTP/MFC) (Figure 4). Manual reference measurements were performed using the in-house PACS (iSite, Philips) and its image analysis toolbox. The inter-reader agreement was determined using the intraclass-correlation-coefficient (online calculator v1.5, Mangold International).

### 2.6. Statistical Analysis

Statistical analysis was performed by SN using GraphpadPrism software (v5.0). For any measure, the absolute differences (Δ_x_) of the respective joint configuration were referenced against the ACL-intact δ_0_-configuration: Δ_x_[ACL-status] = δ_x_[ACL-status]-δ_0_[intact]. Assuming normal distribution, measures were comparatively evaluated using repeated measures ANOVA followed by Tukey’s post-hoc test. Correlations between selected computed 3D and manual 2D measures were quantified using Pearson’s correlation coefficient. Secondary to this study’s exploratory design, numerous statistically relevant variables underwent statistical testing, i.e., eleven computed 3D measures and four manual 2D measures in three ACL conditions and two loading configurations each. In a statistical sense, these measurements may be considered separate experiments. Instead of adhering to statistical formalism by correcting the level of significance for the numerous sub-experiments, a pragmatic and stricter-than-usual level of significance of *p* ≤ 0.01 was chosen to reduce the number of statistically significant, yet clinically (most likely) irrelevant findings while preserving statistical power and decreasing the false-negative rate.

## 3. Results

At δ_0_, the ACL appeared heterogenous with slight intra- and peri-ligamentous signal increases yet without signs of fiber discontinuity or abnormal orientation. Structural and functional integrity of the ACL was confirmed during subsequent arthroscopy.

**Figure 5:**
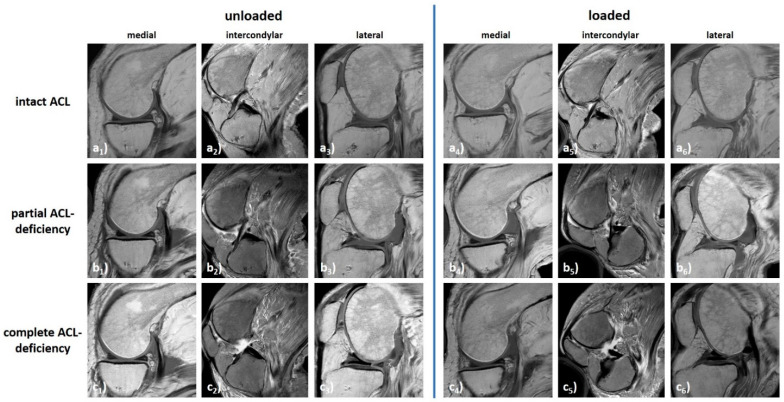
Synopsis of loading-induced changes in a representative knee joint specimen as a function of loading and ACL status. Displayed are T1-weighted sagittal sequences through the centres of the medial (**a_1_**–**c_1_**,**a_4_**–**c_4_**) and lateral (**a_3_**–**c_3_**,**a_6_**–**c_6_**) femorotibial compartments as well as T2-weighted para-sagittal sequences aligned along the ACL (**a_2_**–**c_2_**,**a_5_**–**c_5_**). Unloaded (**a_1_**–**a_3_**,**b_1_**–**b_3_**,**c_1_**–**c_4_**) and loaded configurations (**a_4_**–**a_6_**,**b_4_**–**b_6_**,**c_4_**–**c_6_**). ACL-intact (**a**), partially ACL-deficient (**b**), and completely ACL-deficient conditions (**c**). Same knee joint specimen as in Figure 2, Figure 3 and Figure 4.

The knee joint underwent complex motional changes as a function of ACL status and loading. Qualitatively, more pronounced changes were found in the δ_1_- than δ_0_-configurations, in the lateral than medial femorotibial compartment, and in completely ACL-deficient than partially ACL-deficient and ACL-intact configurations (Figure 5).

**Figure 6:**
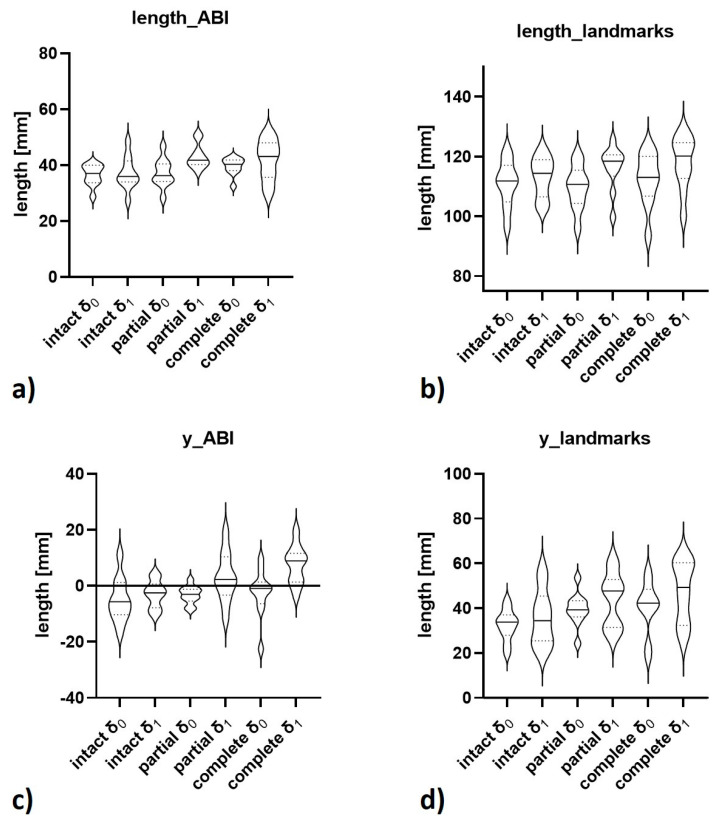
Visualizations of selected computed 3D measures of joint motion as a function of ACL status and loading. Visualized are “length_ABI” (**a**), “length_landmarks” (**b**), “y_ABI” (**c**), and “y_landmarks” (**d**) as violin plots where quartiles are indicated by dotted lines and medians by continuous lines. δ_0_ and δ_1_ refer to the loading configuration and intact, partial, and complete to the ACL condition.

Absolute values of the computed 3D and manual 2D measures as a function of ACL condition and loading configuration are given in Table 2 and visualized in Figure 6. Corresponding post-hoc test results detailing the outcomes of pairwise comparisons are outlined in Supplementary Table S1. Absolute loading-induced differences in these measures versus the ACL-intact δ_0_-configuration and the respective post-hoc test results are detailed in Supplementary Tables S2 and S3, while the absolute differences versus each ACL condition’s δ_0_-configuration are indicated in Supplementary Table S4.

In the ACL-intact configurations, loading-induced motional changes were slight with largest increases along the y-axis (anteroposterior). No computed 3D measure increased beyond means of 3.3 mm and no significant differences between the δ_0_- and δ_1_-configurations were determined.

In the partially ACL-deficient configurations, the joints underwent moderate motional changes under loading. Absolute differences were largest for the vector lengths and their projections along the y-axis, e.g., y_ABI (Δ_1_ = 8.1 ± 8.9 mm) and y_landmarks (Δ_1_ = 11.4 ± 6.4 mm). Numerous significant differences were determined between the ACL-intact δ_0_- and partially ACL-deficient δ_1_-configurations, i.e., length_ABI, z_ABI, and yz-angle.

In the completely ACL-deficient configurations, loading-induced motional changes were even greater, e.g., y_ABI (Δ_1_ = 12.6 ± 6.9 mm) and y_landmarks (Δ_1_ = 14.8 ± 11.0 mm). Changes in vector lengths and vector projections along the other axes were variable. Significant differences were determined for the completely ACL-deficient δ_1_-configuration and other δ_0_- and δ_1_-configurations which primarily involved vector lengths and vector projections along the y-axis. Central-bone-axes-associated angles tended to decrease with loading and increasing ACL injury, yet not significantly.

Manual reference measurements confirmed the findings above with the largest mean differences determined for LTP/LFC, i.e., 3.0 mm [intact]; 6.8 mm [partial]; 7.7 mm [complete], *p* < 0.001. As inter-reader agreement was almost perfect with intraclass-correlation-coefficient values (single scorings, not adjusted) of 0.98 (LMD), 0.99 (LTP/LFC), 0.96 (MMD), and 0.99 (MTP/MFC), both readers’ measurements were pooled. Overall, the lateral femorotibial compartment underwent considerably larger motional changes than the medial compartment.

## 4. Discussion

The most important finding of this study is that stress MRI, combined with comprehensive image post-processing, may be used to parameterize, and quantify, femorotibial kinematics in health and disease by yielding various multidimensional imaging markers of joint stability. Based thereon, partial, and complete ACL injury can be differentiated using both computed 3D and manual 2D measures.

Because morphologic MRI fails in differentiating partial from complete ACL injuries [8,12,13] and stress radiography lacks precision and standardisation [19,20,21,27], consensus is growing that joint stability must be assessed more precisely, reliably, and objectively [10,13,16,17].

Intent to meet this clinical need in a basic research context, this study assessed femorotibial kinematics as a function of ACL status and loading. Through its modular design, our loading device allows precise pressure-controlled loading of joints in various configurations, magnitudes, orientations, and imaging modalities [24]. Emulating the anterior drawer test in the MRI scanner, loading was applied at ≥90° of flexion to reduce loading-induced motional adjustments of the specimens that were only fixed by two counter-bearings. Because of the door stopper-effect of the menisci, i.e., obstructed tibial motion at high flexion angles due to the meniscus, joint loading at 20–30° of flexion—emulating the clinical Lachman test—would certainly increase sensitivity [27,28] in future patient studies. Possible in-situ studies assessing the MRI Lachman test would require entire lower extremities for appropriate confinement.

In clinical practice, even under study conditions, joint flexion during imaging may be reproducible only within a specific range. Aiming for 20°, Noh et al. eventually obtained stress radiographs at 10–30° of flexion while also observing that, once the desired flexion angle was set, it was hardly maintained under loading and affected ATT [21]. Furthermore, ATT is also affected by tibial rotation [29]. Hence, comprehensive image post-processing techniques are a prerequisite to quantify joint flexion and rotation and assess their impact on ATT.

Complex motional changes of the tibia and femur were observed as a function of loading and ACL status. In ACL-intact joints, changes in the anteroposterior dimension of ≤3.6 mm indicate the range of physiological laxity and are well in line with the literature. At 90° of flexion, Kondo et al. determined an increase in ATT of 3.0 ± 1.4 mm under loading of 90 N [29]. Other laboratory and clinical studies confirmed these findings [27,30]. In the partially ACL-deficient joint, vector lengths and projections (along y) increased considerably by 8.1 ± 8.9 mm (y_ABI) and 11.4 ± 6.4 mm (y_landmarks). Increases in ATT with partial ACL injury are well known [9,13,31], yet the exact increase is largely determined by the extent, location, and type of tear, the bundle(s) affected, and the integrity of the remaining ACL fibers. Hence, partial ACL tears are now differentiated as functional and non-functional based on arthroscopic probing [13]. Mechanically solid residual ACL fibres are functional and bring about significantly lower side-to-side differences in ATT (ATT = 4.4 ± 2.4 mm) than non-functional residual ACL fibres (ATT = 7.0 ± 2.5 mm) [13], thereby determining the type of treatment [13,14,15]. Along these lines, our study offers the imaging correlates of functional partial ACL tears as we confirmed functionality of the residual ACL fibres, too. The discrepant ATT values reported in this and other studies must be considered against differences in study designs and measurement methodology. In clinical settings, muscle tone and relaxation, functional deficits, pain and apprehension, concomitant injuries, anaesthesia, and time of examination impact ATT quantification and decrease comparability with laboratory studies [19,27,30]. In completely ACL-deficient joints, loading-induced motional changes were even greater, averaging 12–15 mm (vector projections along y) and 6–8 mm (LTP/LFC and MTP/MFC). This is reflected by literature data, too, that reported mean increases in ATT of 9–15 mm [13,29,30,31].

Overall, statistical variability was substantial despite the clear association of increasing ATT and increasing ACL injury. Some completely ACL-deficient joints remained relatively stable which has been observed before [13,17,22,29,30,31] and may be attributed to variability in passive static stabilizers and ligament restraints (and muscle guarding in patients).

Another relevant aspect relates to rotational knee laxity incurred by ACL deficiency. The ACL restrains internal tibial rotation, which may be a potential diagnostic target. Vassalou et al. reported higher femorotibial angles (between the posterior femoral and tibial condyles) in ACL-deficient as compared to intact knee joints [32], while Polat et al. observed higher tibial tubercle-trochlear groove distances in ACL-injured patients than in controls [33]. Excessive ATT, combined with high internal rotation of the tibia, also alters the lateral collateral ligament’s orientation to be visualized in one coronal slice, which is referred to as the coronal LCL sign [34]. In the present study, rotation is indicated by the xy-angles between central bone axes in the axial plane. Under loading, xy-angles decreased moderately (though not significantly), in particular in partial ACL deficiency, indicating increasing internal rotation. Surprisingly, only slightly increasing internal rotation was found for complete ACL deficiency. Alongside other significant changes beyond the anteroposterior dimension, i.e., increases in z_ABI and decreases in yz-angles, these changes must be considered against the joint’s partial confinement via the tibial medullary extension and yet ill-controlled compensatory motion under motion. These observations are likely due to this study’s experimental setup and remain to be investigated in future clinical studies. This study has numerous limitations. First and foremost, this study is an exploratory study using human cadaveric knee joint specimens. Consequently, the laboratory setting limits direct in-vivo translation of our findings. Beyond the effects of active stabilizers, i.e., hamstring and quadriceps muscles, on knee joint instability, further studies also need to assess the patient-, joint-, loading-, and device-related aspects. Stress radiographic techniques have been in clinical operation for decades and a wide range of loading magnitudes, ranging from 3 to 30 kPa have been applied [19]. Nonetheless, consensus prevails that 15 kP (as used in this study) is mechanically effective, individually tolerated, and diagnostically beneficial [13,24,35,36,37]. However, even when optimized and focused, image acquisition in MRI takes several minutes in contrast to (split) seconds in radiography. Consequently, loading must be applied and upheld for longer time periods, which might affect patient tolerability and increase motion artefacts, and remains to be studied in clinical trials. When realizing the concept’s in-vivo translation, loading efficiency must necessarily be balanced against patient tolerability and comfort, device operability, handling, and safety, joint fixation and stabilization, and measurement validity and reproducibility. Second, our specimens may not be representative of the considerably younger clinical population with stiffer ACLs. Third, tibial rotation was not considered. Alternative approaches quantify ATT in various rotational positions [17,23], yet utilisation by radiologists may be limited due to the specific clinical indication. Fourth, our method was not compared to instrumented laxity measures, clinical gradings or secondary signs of ACL deficiency assessable on standard MRI studies. Any future clinical studies need to include more refined reference measures. In terms of imaging, such references may involve the coronal lateral collateral ligament sign [34], the tibial tubercle-trochlear groove distance [33], and the femorotibial angle [32] that may be compared to the amount of ATT during the MRI anterior drawer test to elucidate the additional potential diagnostic benefit of such stress MRI techniques. Fifth, the transection model failed to emulate the trauma mechanism itself so that associated lesions such as bone bruises or clinically relevant concomitant injuries were not assessed. Sixth, the 3D model relies on exact segmentations that are labour-intensive if performed manually. For streamlined implementation in clinical workflows, segmentation needs to be automated. Seventh, the computed 3D measures only assess global joint motion, while assessment of the medial and lateral compartments would require identification of additional bony landmarks. Eighth, the clinical value and relevance of the 3D measures, particularly vector lengths and angle projections, remains to be determined. While these measures were implemented to be as intuitive and relatable in clinical contexts as possible, alternative approaches to assess tibiofemoral kinematics, e.g., by using coplanar vectors, may be appropriate, too, and remain to be studied. Projective angular measurements are particularly prone to cross-plane effects because coronal, sagittal, and axial alignment affect these measures. For example, projected xy-angles (corresponding to the axial projection) are influenced by valgus and varus alignment and, therefore, these angles do not merely indicate internal and external rotation. These aspects complicate their interpretation and remain to be studied against conventional diagnostic measures of internal or external rotation. Additionally, vector projections are affected by joint flexion and position that were not fully standardized between the separate measurements resulting in inter-measurement variability. Consequently, evaluation of loading-induced quantitative changes seems more accurate in the same ACL condition and between δ_0_ and δ_1_; however, such intra-conditional references may not be as intuitive as comparisons to the ACL-intact δ_0_-configuration. Ninth, specimens were fresh frozen at −20°C for days to weeks. Freezing and thawing alters ACL ultrastructure and, consequently, biomechanical properties [38]. Even though their exact effects remain unclear, storage conditions may have differentially altered mechanical properties of joints’ secondary stabilizers. Consequently, this systematic error needs to be considered when undertaking the eventual clinical translation. Tenth, the extended and partially redundant imaging protocol inflated overall magnet time and is not suitable for clinical use. A more focused protocol for future clinical studies that includes T1-weighted (and possibly, proton density-weighted fat-saturated) sequences will reduce magnet time to be adequate for clinical workflows.

## 5. Conclusions

In conclusion, this study is the first of its kind to implement the clinical anterior drawer test (for the assessment of knee joint stability) in an in-situ MRI setting and to study the effects of standardized pressure-controlled loading on the MRI appearance of intact, partially, and completely ACL-deficient human knee joints using standardized arthroscopic transections. Beyond providing normative data on femorotibial kinematics as a function of ACL status and loading in human cadaveric specimens, this study uses the MRI anterior drawer test to obtain imaging markers of joint stability. By adding mechanical loading to the MRI protocol, ligaments may be diagnostically evaluated beyond morphology and in more functional contexts with potential applications in differentiating partial and complete ACL injuries, monitoring treatment outcomes, and comparing the efficacy of surgical procedures.

## Figures and Tables

**Table 1:** Acquisition parameters of MR sequences. Abbreviations are PDW-proton density-weighted, fs-fat-saturated, TSE-turbospin-echo, cor-coronal, (para)ax-(para)axial, (para)sag-(para)sagittal, SPAIR-Spectral Attenuated Inversion Recovery, n/a-not applicable. (*) indicates that both angulations were strictly sagittal and axial to the course of the ACL, i.e., aligned along its long axis, and hence parasagittal and paraxial to the joint. Please note that the sagittal T1-weighted sequence was used for performing manual segmentations, computing 3D joint models, and determining 3D measures of joint motion.

Sequence Parameters	PDW fs	PDW fs	PDW fs	T1	T2
**Sequence type**	**2D TSE**	**2D TSE**	**2D TSE**	**2D TSE**	**2D TSE**
**Orientation**	cor	ax	sag	sag	parasag & parax (*)
**Type of fat saturation**	SPAIR	SPAIR	SPAIR	n/a	n/a
**Repetition time [ms]**	4495	4776	4928	671	3000
**Echo time [ms]**	30	30	30	9	80
**Turbo spin-echo factor**	13	13	15	3	14
**Field of view [mm]**	160 × 160	160 × 160	160 × 160	160 × 160	160 × 160
**Acquisition matrix [pixels]**	400 × 300	400 × 312	352 × 255	368 × 317	352 × 295
**Reconstruction matrix [pixels]**	512 × 512	512 × 512	512 × 512	448 × 448	512 × 512
**Scan percentage [%]**	79.4	79.4	79.3	86.1	85.0
**Flip angle [°]**	90	90	90	90	90
**Number of signal averages [n]**	1	1	1	1	1
**Slices [n]**	31	33	26	30	7
**Slice thickness/gap [mm]**	3.0/0.3	3.0/0.3	3.0/0.3	3.0/0.3	3.0/0.3
**Duration [min:sec]**	04:39	03:59	03:27	06:40	07:30

**Table 2:** Absolute values of computed 3D and manual 2D measures of joint motion as a function of ACL status and loading. Data are mean ± standard deviation. The 3D measures were determined based on segmentation outlines, anatomic landmarks, bone axes, and computed fixpoints and used to quantify the knee joint specimens’ motion along the x-axis (mediolateral), y-axis (anteroposterior), and z-axis (craniocaudal) using a 3D Cartesian coordinate system. Moreover, 3D Euclidean vectors between the femoral and tibial axis-bone-intersections (i.e., fABI to tABI, “vector_ABI”) and two anatomic landmarks (i.e., centre of tibial tuberosity to tip of the cartilage-covered trochlea, “vector_landmarks”) were quantified in length (“length_ABI”, “length_landmarks”) and vector projections along the x-axis (“x_ABI”, “x_landmarks”), y-axis (“y_ABI”, “y_landmarks”), and z-axis (“z_ABI”, “z_landmarks”). Angles between the central bone axes (“xz-angle”, “yz-angle”, “xy-angle”) were determined following planar projection. The manual measures were determined on mid-sagittal slices of the medial and lateral femorotibial compartments by relating tibial motion to the menisci (LMD, MMD) or the femoral condyles (LTP/LFC, MTP/MFC). Note that the manual 2D measurements by the two readers were pooled. Repeated measures ANOVA was used to test for statistical significance between the measures of each ACL condition and joint configuration, i.e., ACL-intact, partially, or completely ACL-deficient and unloaded (δ_0_) or loaded (δ_1_). Statistically significant findings are indicated in bold type and sequentially numbered in square brackets. The corresponding post-hoc results are detailed in Supplementary Table S1.

Category of Measure	Measure [Unit]	Description of Measure	Intact		Partial ACL Deficiency		Complete ACL Deficiency		*p*-Value
			δ_0_	δ_1_	δ_0_	δ_1_	δ_0_	δ_1_	
**Computed 3D Measures**	**length_ABI [mm]**	length of vector_ABI	36.4 ± 3.8	37.2 ± 5.9	37.2 ± 5.1	43.1 ± 4.4	39.6 ± 3.1	41.8 ± 7.3	**<0.001** [1]
	**x_ABI [mm]**	projected length of vector_ABI along x-axis (mediolateral)	−2.3 ± 8.7	−1.8 ± 4.7	−3.5 ± 4.6	−5.4 ± 6.0	−5.0 ± 6.5	−2.0 ± 6.4	0.579
	**y_ABI [mm]**	projected length of vector_ABI along y-axis (anteroposterior)	−5.0 ± 7.9	−3.0 ± 4.9	−3.3 ± 3.2	3.1 ± 8.9	−2.6 ± 8.6	7.6 ± 6.8	**<0.001** [2]
	**z_ABI [mm]**	projected length of vector_ABI along z-axis (craniocaudal)	34.0 ± 5.5	36.5 ± 5.9	36.5 ± 4.9	41.4 ± 4.1	37.3 ± 7.6	40.2 ± 6.4	**<0.001** [3]
	**length_landmarks [mm]**	length of vector_landmarks	110.9 ± 7.8	113.2 ± 6.8	109.7 ± 7.3	116.3 ± 7.4	112.4 ± 8.9	118.0 ± 8.9	**<0.001** [4]
	**x_landmarks [mm]**	projected length of vector_landmarks along x-axis (mediolateral)	7.3 ± 5.1	7.5 ± 3.7	5.2 ± 3.7	5.4 ± 4.4	7.3 ± 6.4	5.0 ± 3.6	0.644
	**y_landmarks [mm]**	projected length of vector_landmarks along y-axis (anteroposterior)	32.5 ± 7.0	35.8 ± 12.6	39.5 ± 7.5	43.9 ± 12.1	40.5 ± 11.9	47.3 ± 13.7	**0.003** [5]
	**z_landmarks [mm]**	projected length of vector_landmarks along z-axis (craniocaudal)	105.5 ± 7.1	106.4 ± 5.8	102.0 ± 5.7	106.9 ± 7.7	103.9 ± 7.6	107.3 ± 7.3	0.110
	**xz-angle [°]**	projected angle of central bone axes on xz-plane (coronal)	143.6 ± 15.0	140.7 ± 24.7	138.0 ± 15.2	130.8 ± 22.1	137.0 ± 28.1	117.5 ± 18.6	0.016
	**yz-angle [°]**	projected angle of central bone axes on yz-plane (sagittal)	109.5 ± 11.7	103.3 ± 9.7	108.8 ± 10.3	98.0 ± 5.1	101.5 ± 12.1	101.2 ± 8.5	**<0.001** [6]
	**xy-angle [°]**	projected angle of central bone axes on xy-plane (axial)	34.9 ± 21.4	29.5 ± 25.6	39.0 ± 29.6	25.5 ± 16.3	28.7 ± 23.7	26.2 ± 20.6	0.723
**Manual 2D Measures**	**LMD [mm]**	distance between posterior horn (lateral meniscus) and posterior margin (tibial plateau)	4.1 ± 1.6	0.9 ± 2.1	4.0 ± 2.9	0.1 ± 2.1	2.7 ± 2.7	−0.2 ± 3.0	**0.001** [7]
	**LTP/LFC [mm]**	distance between femoral condyle (lateral) and posterior margin (tibial plateau)	−3.0 ± 2.7	−6.0 ± 5.1	−4.0 ± 3.8	−9.9 ± 3.6	−5.4 ± 3.8	−10.8 ± 4.1	0.302
	**MMD [mm]**	distance between posterior horn (medial meniscus) and posterior margin (tibial plateau)	3.7 ± 1.4	1.8 ± 2.4	3.1 ± 2.2	0.3 ± 1.7	1.8 ± 1.0	−0.3 ± 1.3	**<0.001** [8]
	**MTP/MFC [mm]**	distance between femoral condyle (medial) and posterior margin (tibial plateau)	1.2 ± 1.8	−0.7 ± 3.4	0.3 ± 4.3	−2.4 ± 2.4	−1.4 ± 2.0	−4.6 ± 2.4	**<0.001** [9]

## Data Availability

The data not contained in the manuscript or supplementary material is available from the corresponding author upon reasonable request.

## Supplementary Materials

The following are available online at https://www.mdpi.com/article/10.3390/diagnostics11061035/s1, Supplementary File S1: technical description and detailed visualization of the post-processing methodology, Supplementary Table S1: post-hoc details of computed 3D and manual 2D measures. Supplementary Table S2: absolute loading-induced differences of computed 3D measures and manual 2D measures of joint motion as a function of ACL status and loading. Supplementary Table S3: post-hoc details of absolute differences of computed 3D and manual 2D measures. Supplementary Table S4: absolute differences between loaded and unloaded configurations as a function of ACL condition.

## References

[1] Majewski M., Susanne H., Klaus S. (2006). Epidemiology of athletic knee injuries: A 10-year study. Knee.

[2] Bollen S. (2000). Epidemiology of knee injuries: Diagnosis and triage. Br. J. Sports Med..

[3] Granan L.P., Forssblad M., Lind M., Engebretsen L. (2009). The Scandinavian ACL registries 2004–2007: Baseline epidemiology. Acta Orthop..

[4] Shakoor D., Guermazi A., Kijowski R., Fritz J., Roemer F.W., Jalali-Farahani S., Demehri S. (2019). Cruciate ligament injuries of the knee: A meta-analysis of the diagnostic performance of 3D MRI. J. Magn. Reson. Imaging.

[5] Chen W.-T., Shih T.-F., Tu H.-Y., Chen R.-C., Shau W.-Y. (2002). Partial and complete tear of the anterior cruciate ligament: Direct and indirect MR signs. Acta Radiol..

[6] Umans H., Wimpfheimer O., Haramati N., Applbaum Y., Adler M., Bosco J. (1995). Diagnosis of partial tears of the anterior cruciate ligament of the knee: Value of MR imaging. AJR Am. J. Roentgenol..

[7] Lefevre N., Naouri J., Bohu Y., Klouche S., Herman S. (2014). Partial tears of the anterior cruciate ligament: Diagnostic performance of isotropic three-dimensional fast spin echo (3D-FSE-Cube) MRI. Eur. J. Orthop. Surg. Traumatol..

[8] Ng A.W., Griffith J.F., Hung E.H., Law K.Y., Yung P.S. (2013). MRI diagnosis of ACL bundle tears: Value of oblique axial imaging. Skelet. Radiol..

[9] Sonnery-Cottet B., Colombet P. (2016). Partial tears of the anterior cruciate ligament. Orthop. Traumatol. Surg. Res..

[10] Ohashi B., Ward J., Araujo P., Kfuri M., Pereira H., Espregueira-Mendes J., Musahl V. (2015). Partial Anterior Cruciate Ligament Ruptures: Knee laxity measurements and pivot shift. Sports Injuries: Prevention, Diagnosis, Treatment and Rehabilitation.

[11] Noyes F., Mooar L., Moorman 3rd C., McGinniss G. (1989). Partial tears of the anterior cruciate ligament. Progression to complete ligament deficiency. J. Bone Jt. Surg. Br. Vol..

[12] Van Dyck P., De Smet E., Veryser J., Lambrecht V., Gielen J.L., Vanhoenacker F.M., Dossche L., Parizel P.M. (2012). Partial tear of the anterior cruciate ligament of the knee: Injury patterns on MR imaging. Knee Surg. Sports Traumatol. Arthrosc..

[13] Dejour D., Ntagiopoulos P.G., Saggin P.R., Panisset J.-C. (2013). The diagnostic value of clinical tests, magnetic resonance imaging, and instrumented laxity in the differentiation of complete versus partial anterior cruciate ligament tears. Arthrosc. J. Arthrosc. Relat. Surg..

[14] DeFranco M.J., Bach B.R. (2009). A comprehensive review of partial anterior cruciate ligament tears. J. Bone Jt. Surg. Am..

[15] Siebold R., Fu F.H. (2008). Assessment and augmentation of symptomatic anteromedial or posterolateral bundle tears of the anterior cruciate ligament. Arthroscopy.

[16] Al-Dadah O., Shepstone L., Marshall T.J., Donell S.T. (2011). Secondary signs on static stress MRI in anterior cruciate ligament rupture. Knee.

[17] Espregueira-Mendes J., Andrade R., Leal A., Pereira H., Skaf A., Rodrigues-Gomes S., Oliveira J.M., Reis R.L., Pereira R. (2017). Global rotation has high sensitivity in ACL lesions within stress MRI. Knee Surg. Sports Traumatol. Arthrosc..

[18] Temponi E.F., de Carvalho Júnior L.H., Sonnery-Cottet B., Chambat P. (2015). Partial tearing of the anterior cruciate ligament: Diagnosis and treatment. Rev. Bras. Ortop..

[19] James E.W., Williams B.T., LaPrade R.F. (2014). Stress radiography for the diagnosis of knee ligament injuries: A systematic review. Clin. Orthop. Relat. Res..

[20] Lee Y.S., Han S.H., Jo J., Kwak K.-s., Nha K.W., Kim J.H. (2011). Comparison of 5 different methods for measuring stress radiographs to improve reproducibility during the evaluation of knee instability. Am. J. Sports Med..

[21] Noh J.H., Nam W.D., Roh Y.H. (2019). Anterior tibial displacement on preoperative stress radiography of ACL-injured knee depending on knee flexion angle. Knee Surg. Relat. Res..

[22] Schneider M., Pinskerova V., Breusch S., Noe V., Freeman M. (2006). Observations of normal and ACL-deficient knee joints after stress MRI. Der Orthop..

[23] Espregueira-Mendes J., Pereira H., Sevivas N., Passos C., Vasconcelos J.C., Monteiro A., Oliveira J.M., Reis R.L. (2012). Assessment of rotatory laxity in anterior cruciate ligament-deficient knees using magnetic resonance imaging with Porto-knee testing device. Knee Surg. Sports Traumatol. Arthrosc..

[24] Said O., Schock J., Kramer N., Thuring J., Hitpass L., Schad P., Kuhl C., Abrar D., Truhn D., Nebelung S. (2020). An MRI-compatible varus-valgus loading device for whole-knee joint functionality assessment based on compartmental compression: A proof-of-concept study. Magn. Reson. Mater. Phys. Biol. Med..

[25] Yoon J.P., Yoo J.H., Chang C.B., Kim S.J., Choi J.Y., Yi J.H., Kim T.K. (2013). Prediction of chronicity of anterior cruciate ligament tear using MRI findings. Clin. Orthop. Surg..

[26] Yushkevich P.A., Piven J., Hazlett H.C., Smith R.G., Ho S., Gee J.C., Gerig G. (2006). User-guided 3D active contour segmentation of anatomical structures: Significantly improved efficiency and reliability. NeuroImage.

[27] Lee H.-J., Park Y.-B., Kim S.H. (2019). Diagnostic value of stress radiography and arthrometer measurement for anterior instability in anterior cruciate ligament injured knees at different knee flexion position. Arthrosc. J. Arthrosc. Relat. Surg..

[28] Katz J.W., Fingeroth R.J. (1986). The diagnostic accuracy of ruptures of the anterior cruciate ligament comparing the Lachman test, the anterior drawer sign, and the pivot shift test in acute and chronic knee injuries. Am. J. Sports Med..

[29] Kondo E., Merican A.M., Yasuda K., Amis A.A. (2014). Biomechanical analysis of knee laxity with isolated anteromedial or posterolateral bundle–deficient anterior cruciate ligament. Arthrosc. J. Arthrosc. Relat. Surg..

[30] Loh J.C., Fukuda Y., Tsuda E., Steadman R.J., Fu F.H., Woo S.L. (2003). Knee stability and graft function following anterior cruciate ligament reconstruction: Comparison between 11 o’clock and 10 o’clock femoral tunnel placement. Arthrosc. J. Arthrosc. Relat. Surg..

[31] Zantop T., Herbort M., Raschke M.J., Fu F.H., Petersen W. (2007). The Role of the Anteromedial and Posterolateral Bundles of the Anterior Cruciate Ligament in Anterior Tibial Translation and Internal Rotation. Am. J. Sports Med..

[32] Vassalou E.E., Klontzas M.E., Kouvidis G.K., Matalliotaki P.I., Karantanas A.H. (2016). Rotational knee laxity in anterior cruciate ligament deficiency: An additional secondary sign on MRI. Am. J. Roentgenol..

[33] Polat A.E., Polat B., Gürpınar T., Sarı E., Çarkçı E., Erler K. (2020). Tibial tubercle–trochlear groove (TT–TG) distance is a reliable measurement of increased rotational laxity in the knee with an anterior cruciate ligament injury. Knee.

[34] Mitchell B.C., Siow M.Y., Bastrom T., Bomar J.D., Pennock A.T., Parvaresh K., Edmonds E.W. (2021). Coronal lateral collateral ligament sign: A novel magnetic resonance imaging sign for identifying anterior cruciate ligament–deficient knees in adolescents and summarizing the extent of anterior tibial translation and femorotibial internal rotation. Am. J. Sports Med..

[35] Panisset J.-C., Ntagiopoulos P.-G., Saggin P., Dejour D. (2012). A comparison of Telos™ stress radiography versus Rolimeter™ in the diagnosis of different patterns of anterior cruciate ligament tears. Orthop. Traumatol. Surg. Res..

[36] Garces G., Perdomo E., Guerra A., Cabrera-Bonilla R. (1995). Stress radiography in the diagnosis of anterior cruciate ligament deficiency. Int. Orthop..

[37] Rijke A.M., Tegtmeyer C., Weiland D., McCue F. (1987). Stress examination of the cruciate ligaments: A radiologic Lachman test. Radiology.

[38] Lansdown D.A., Riff A.J., Meadows M., Yanke A.B., Bach B.R. (2017). What factors influence the biomechanical properties of allograft tissue for ACL reconstruction? A systematic review. Clin. Orthop. Relat. Res..

