# Supplementary material for: Seeing Beyond Morphology-Standardized Stress MRI to Assess Human Knee Joint Instability
Source: Diagnostics (Basel). 2021 Jun 4;11(6):1035. doi: 10.3390/diagnostics11061035 (PMC8226919; doi:10.3390/diagnostics11061035)
Supplement: Supplementary file 1 [file diagnostics-11-01035-s001.zip › diagnostics-1219686-supplementary.pdf]

Supplementary Material

Title: Seeing Beyond Morphology – Standardized Stress MRI to Assess Human Knee Joint Instability

Authors: Eva-Maria Winkelmeyer, Justus Schock, Lena Marie Wollschläger, Philipp Schad, Marc Sebastian Huppertz, Niklas Kotowski, Andreas Prescher, Christiane Kuhl, Daniel Truhn, Sven Nebelung

Supplementary Text 1: Technical description and detailed visualization of the post-processing methodology.

The image post-processing methodology involved manual image segmentations, manual identification and registration of anatomic landmarks and axes, computational identification of additional fixpoints, and subsequent parameterization and calculation of motional changes of the femur and tibia as a function of loading and anterior cruciate ligament (ACL)-status.

1) Manual Segmentations of Femoral and Tibial Bone Contours

The femoral and tibial bone contours of the unloaded and loaded configurations of the three ACL conditions (i.e. intact, partially, and completely ACL-deficient) were segmented by EMW (medical student, 2 years of experience in musculoskeletal imaging) using the semiautomatic segmentation function of ITK-SNAP 3.8 software (Cognitica) (https://www.itksnap.org).[30] To this end, the bone contours were manually delineated for each measurement on sagittal T1-weighted images (**Figure SF 1**).

In addition, automatic pre-processing and harmonization procedures were implemented in Python (v3.6.5, Python Software Foundation) to realize consistent segmentation outlines while discarding mis-segmented voxels. Connected-component analysis was performed to automatically discard all voxels that were not in contact with the manual segmentation outlines. Correspondingly, all voxels that were located within the segmentation outlines but had -erroneously- not been included were then filled and labelled appropriately. Once these procedures were completed, segmentation outlines were comparatively re-evaluated against the original MR images.

2) Identification and Registration of Anatomic Landmarks and Axes

Furthermore, the central bone axes of the femur and tibia as well as distinct femoral and tibial anatomic landmarks were manually identified and registered in ITK-SNAP using the software's line-and-ruler tool and multi-viewer capabilities. For each joint in each configuration, the following coordinates were registered:

- i) centre of proximal femoral diaphysis (**Figure SF 2a**),
- ii) centre of distal femoral diaphysis (**Figure SF 2b**),

- iii) centre of proximal tibial diaphysis (**Figure SF 2c**),
- iv) centre of distal tibial diaphysis (**Figure SF 2d**),
- v) centre of tibial tuberosity (**Figure SF 2e**), and
- vi) the tip of the cartilage-covered femoral trochlea (**Figure SF 2f**).

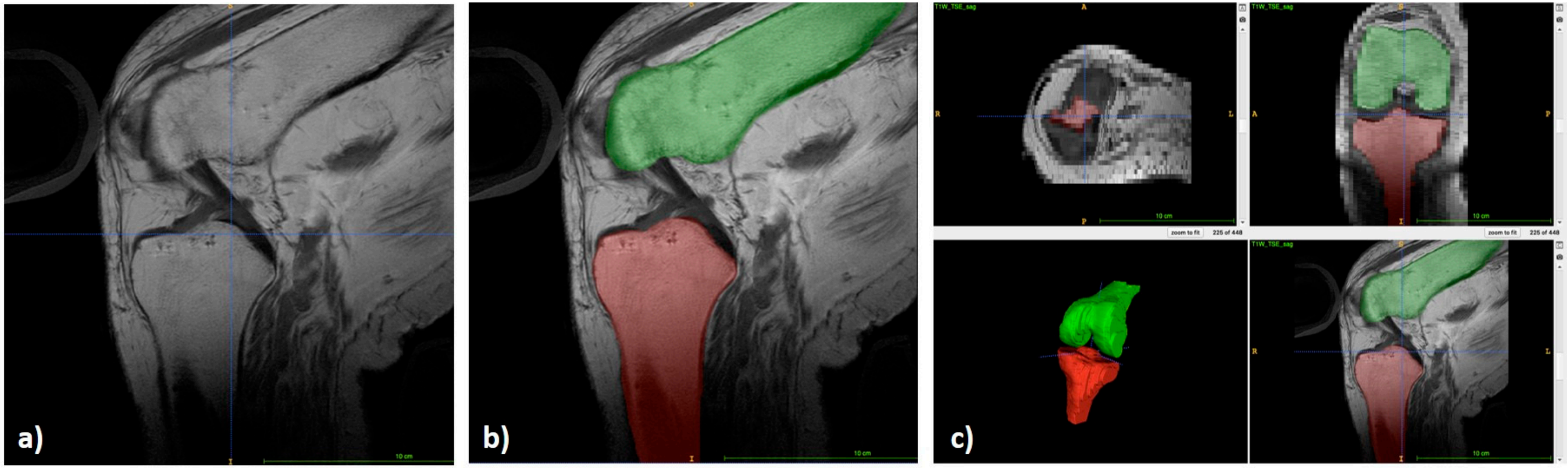

Figure SF 1: Details of the segmentation procedure.
a) Original T1-weighted sagittal image. b) Corresponding manual segmentations of the femur (green) and tibia (red). Using the active contour segmentation functionality implemented in the software (ITK-SNAP, v3.8, Cognitica, Philadelphia, PA, US), pre-segmentations of the bone outlines were obtained and manually corrected on all slices per knee joint specimen and configuration. c) Corresponding multiplanar reconstructions (axial [upper left], coronal [upper right] and 3D bone model [lower left]).

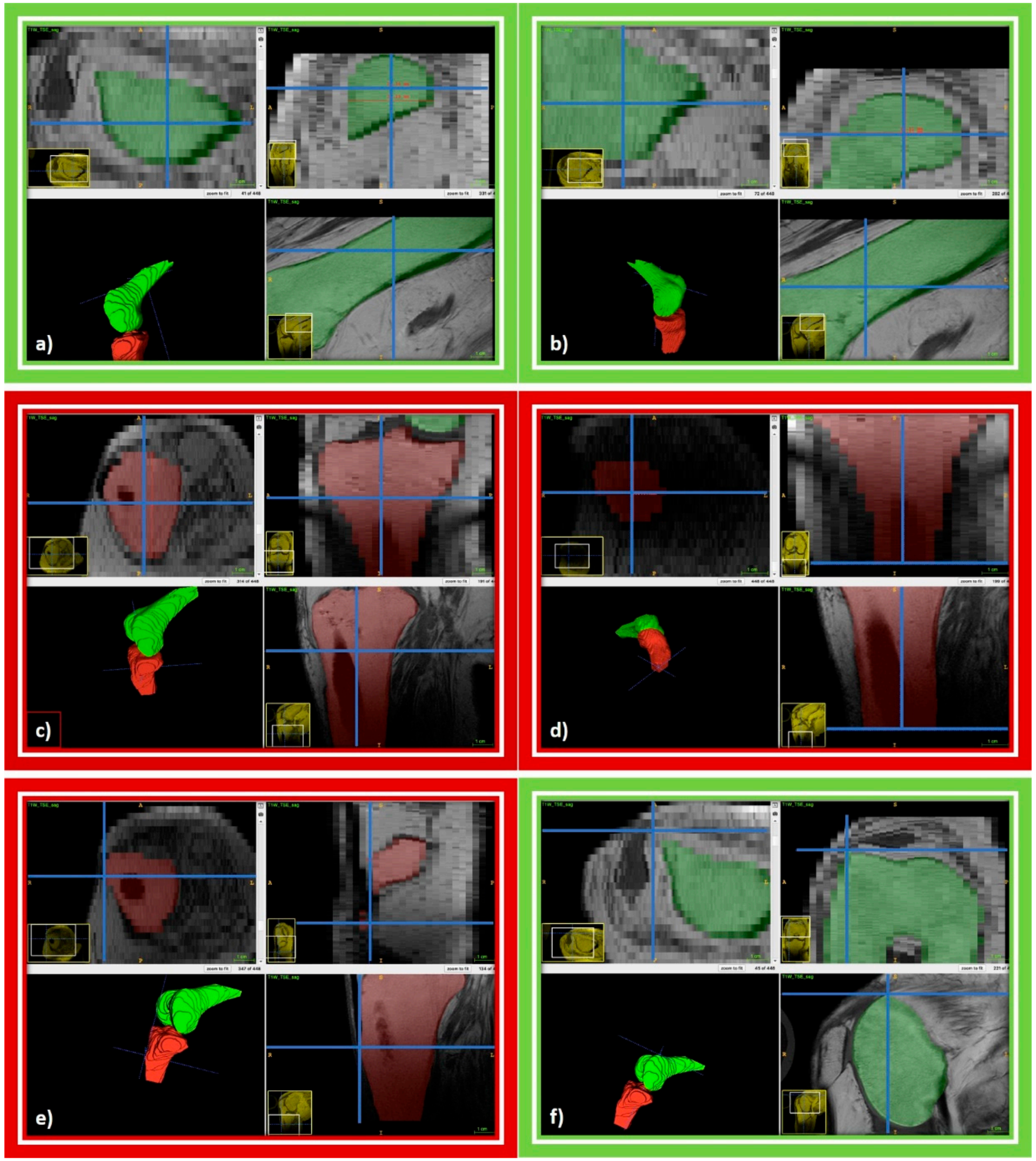

Figure SF 2: Identification of anatomic landmarks to define the central femoral (a, b) and tibial (c, d) bone axes and the additional femoral (e) and tibial (f) anatomic landmarks.
a) Centre of proximal femoral diaphysis: For the registration of these coordinates, the centre of the bone shaft was identified on the most proximal, still fully visualized cross-section of the femur. Using the line-and-ruler tool (ITK-SNAP), the coordinates were determined along the mediolateral and anteroposterior dimensions.
b) Centre of distal femoral diaphysis: Analogously, the coordinates of the centre of the distalmost femoral diaphysis were registered.
c) Centre of proximal tibial diaphysis: Analogously, the coordinates of the centre of the proximalmost tibial diaphysis were registered.
d) Centre of distal tibial diaphysis: Analogously, the coordinates of the centre of the distalmost tibial diaphysis were registered.
e) Centre of tibial tuberosity: For the registration of these coordinates, the most prominent anterior extension of the tibial tuberosity was identified.
f) Tip of cartilage-covered femoral trochlea: Analogously, the most proximal extension of the femoral trochlea still covered by hyaline cartilage was identified.
Selected coordinates are indicated by the cross-sections of thick blue lines, while auxiliary measurements are indicated by thin red lines. Coordinates and landmarks on the femoral side are framed in green, while, correspondingly, coordinates and landmarks on the tibial side are framed in red.

3) Quality Control

All segmentation outlines, axes, and anatomic landmarks were reviewed for consistency and accuracy by SN (clinical radiologist, 8 years of experience in musculoskeletal imaging) and corrected, if needed.

4) Computation of Additional Fixpoints

For more comprehensive analysis of knee joint motion, two additional fixpoints were computed as the intersections of the extended central bone axes, i.e. between coordinates i) and ii) and between coordinates iii) and iv), and the segmentation outlines at the articulating surfaces of the distal femur and the proximal tibia. These fixpoints provided the femoral and tibial axis-bone-intersections.

5) Parameterization of Knee Joint Motion

A 3D knee joint motion model was implemented on the basis of these data to parameterize femoral and tibial motion as a function of loading and ACL status.

3D Euclidean vectors were determined between the anatomic landmarks, i.e. between coordinates v) and vi) (vector 1, "vector_landmarks"), and between the computed femoral and tibial axis-bone-intersections (vector 2, "vector_ABI"). For these vectors, the following measures were obtained:

- vector lengths (**"length_landmarks"**, **"length_ABI"**),
- vector projections on the Cartesian axis x (**"x_landmarks"**, **"x_ABI"**), i.e. the vector length following projection on the x-axis, corresponding to the mediolateral dimension,
- vector projections on the Cartesian axis y (**"y_landmarks"**, **"y_ABI"**), i.e. the vector length following projection on the y-axis, corresponding to the anteroposterior dimension,
- vector projections on the Cartesian axis z (**"z_landmarks"**, **"z_ABI"**), i.e. the vector length following projection on the z-axis, corresponding to the craniocaudal dimension,
- angles between the femoral and tibial central bone axes of the femur and tibia were determined as their projections on the xz-plane (**"xz-angle"**, corresponding to the angle in the coronal plane), yz-plane (**"yz-angle"**, sagittal plane), and xy-plane (**"xy-angle"**, axial plane).

6) Quantification of Knee Joint Motion

The knee joint's overall translation in the mediolateral, anteroposterior, and craniocaudal dimensions were subsequently quantified based on these 3D measures.

- For **vector lengths**, small values indicate close proximity of the two respective anatomic landmarks or computed fixpoints, while higher values indicate greater distance.

- For **vector projections on the x-axis**, positive values indicate a more lateral position of the femoral as compared to the tibial reference point, so that the vector runs from proximal-lateral to distal-medial.
- For **vector projections on the y-axis**, positive values indicate a more posterior position of the femoral as compared to the tibial reference point, so that the vector runs from proximal-posterior to distal-anterior.
- For **vector projections on the z-axis**, positive values indicate a more proximal position of the femoral as compared to the tibial reference point (anatomic!), so that the vector runs from proximal to distal.
- For **xz-angles**, large values close to 180° indicate nearly parallel orientations of the femoral and tibial central bone axes, while values close to 90° indicate nearly right-angled orientations. By convention, xz-angles were defined as the medial-sided angles between the central bone axes in the coronal plane.
- For **yz-angles**, large values close to 180° indicate nearly full extension of the knee joint, while lower angles between 180° and 90° indicate increasing knee joint flexion angles and an yz-angle of 90° perpendicular orientation of the central bone axes in the sagittal plane.
- For **xy-angles**, values close to 0° indicate nearly parallel orientations of the central bone axes in the axial plane. By convention, xy-angles were defined as the medial-sided angles between the central bone axes in the axial plane. Consequently, low-to-high xy-angles indicate the continuous spectrum from internal to external rotation with values close to 90° indicating neutral rotation.

<u>7) Conversion of Measures</u>

Initial voxel-wise vector measurements were converted to mm based on voxel size of 0.36 x 0.36 x 3.0 [mm^3] and an inter-slice gap of 0.3 mm.

<u>8) Reproducibility Assessment</u>

Intra-reader reproducibility in defining the coordinates i) - vi) was assessed on two knee joints and their six configurations each, totalling 12 configurations. In a blinded manner, both measurements were conducted by EW eight weeks apart by registering the respective landmarks' coordinates and by calculating their overall deviation, analogous to the vector lengths (**Table SF1**).

Table SF 1: Mean inter-measurement deviations of six manually defined coordinates corresponding to central bone axes and anatomic landmarks.

Coordinate	Description	Inter-Measurement Deviation [mm]
**i)**	centre of the most proximal femoral diaphysis	1.18 ± 0.95
**ii)**	centre of the most distal femoral diaphysis	1.47 ± 0.80
**iii)**	centre of the most proximal tibial diaphysis	0.94 ± 0.63
**iv)**	centre of the most distal tibial diaphysis	0.94 ± 0.63
**v)**	centre of the tibial tuberosity	1.67 ± 0.91
**vi)**	tip of cartilage-covered femoral trochlea	0.73 ± 0.44

Supplementary Table S1: Post-hoc details of computed 3D and manual 2D measures. Following repeated measures ANOVA, Tukey's multiple comparisons test was performed wherever appropriate to detail statistical significance of the pair-wise comparisons of the measures at each joint configuration, i.e., ACL-intact, partially (partACL) or completely ACL-deficient (compACL), and unloaded (δ_0) or loaded (δ_1). Numbers in square brackets indicate the post-hoc details of the measures given in **Table 2**. Level of significance was set to $p \leq 0.01$ and further stratified into $0.01 \leq p < 0.001$ (**) and $p \leq 0.001$ (***).

**Tukey's post-hoc test**	**Computed 3D Measures**						**Manual 2D Measures**		
	**length_ABI [1]**	**y_ABI [2]**	**z_ABI [3]**	**length_landmarks [4]**	**y_landmarks [5]**	**angle_yz [6]**	**LMD [7]**	**MMD [8]**	**MTP/MFC [9]**
**intact δ_0 vs. intact δ_1**	ns	ns	ns	ns	ns	ns	*	ns	ns
**intact δ_0 vs. partACL δ_0**	ns	ns	ns	ns	ns	ns	ns	ns	ns
**intact δ_0 vs. partACL δ_1**	**	ns	**	ns	ns	**	**	***	*
**intact δ_0 vs. compACL δ_0**	ns	ns	ns	ns	ns	ns	ns	ns	ns
**intact δ_0 vs. compACL δ_1**	ns	***	**	**	**	ns	***	***	***
**intact δ_1 vs. partACL δ_0**	ns	ns	ns	ns	ns	ns	*	ns	ns
**intact δ_1 vs. partACL δ1**	**	ns	ns	ns	ns	ns	ns	ns	ns
**intact δ_1 vs. compACL δ_0**	ns	ns	ns	ns	ns	ns	ns	ns	ns
**intact δ_1 vs. compACL δ_1**	ns	***	ns	ns	ns	ns	ns	ns	**
**partACL δ_0 vs. partACL δ_1**	**	ns	ns	**	ns	**	**	**	ns
**partACL δ_0 vs. compACL δ_0**	ns	ns	ns	ns	ns	ns	ns	ns	ns
**partACL δ_0 vs. compACL δ_1**	ns	***	ns	***	ns	ns	ns	***	***
**partACL δ_1 vs. compACL δ_0**	ns	ns	ns	ns	ns	ns	ns	ns	ns
**partACL δ_1 vs. compACL δ_1**	ns	ns	ns	ns	ns	ns	ns	ns	ns
**compACL δ_0 vs. compACL δ_1**	ns	**	ns	ns	ns	ns	ns	ns	ns

Supplementary Table S2: Absolute loading-induced differences of computed 3D measures and manual 2D measures of joint motion as a function of ACL status and loading. For any measure, the absolute differences (Δ_x) of the respective ACL condition and joint configuration, i.e. ACL-intact, partially or completely ACL-deficient, and unloaded (δ_0) or loaded (δ_1), were referenced against the ACL-intact δ_0-configuration and calculated as Δ_x[ACL condition]=δ_x[ACL condition]-δ_0[intact]. Data are means ± standard deviations. Repeated measures ANOVA was used to test for statistical significance between the absolute differences. Statistically significant findings are indicated in bold type and sequentially numbered in square brackets; the corresponding post-hoc details are detailed in **Supplementary Table S3**. Note that the manual 2D measurements by the two readers were pooled. Please refer to **Table 2** for an explanation of the abbreviations.

Category of Measure	Measure [Unit]	absolute differences Δ (vs. intact unloaded [δ_0])					
		**intact**	**partial ACL deficiency**		**complete ACL deficiency**		**p-value**
		**loaded (δ_1)**	**unloaded (δ_0)**	**loaded (δ_1)**	**unloaded (δ_0)**	**loaded (δ_1)**	
Computed 3D Measure (Axis-Bone-Intersections)	**length_ABI [mm]**	0.8 ± 5.0	0.8 ± 2.3	6.7 ± 3.8	3.1 ± 2.3	5.4 ± 4.8	**0.004 [1]**
	**x_ABI [mm]**	0.6 ± 7.0	-1.2 ± 7.1	-3.1 ± 10.6	-2.6 ± 11.7	0.3 ± 9.9	0.381
	**y_ABI [mm]**	2.0 ± 9.1	1.7 ± 6.8	8.1 ± 8.9	2.4 ± 8.7	12.6 ± 6.9	**< 0.001 [2]**
	**z_ABI [mm]**	2.4 ± 6.1	2.5 ± 2.9	7.4 ± 4.2	3.2 ± 5.1	6.2 ± 3.7	0.024
Computed 3D Measure (Anatomic Landmarks)	**length_landmarks [mm]**	2.2 ± 4.3	-1.2 ± 5.8	5.4 ± 4.1	1.5 ± 2.7	7.1 ± 3.3	**< 0.001 [3]**
	**x_landmarks [mm]**	0.2 ± 5.1	-2.1 ± 6.5	-1.9 ± 7.4	0.0 ± 8.8	-2.3 ± 6.8	0.562
	**y_landmarks [mm]**	3.3 ± 11.2	7.0 ± 7.6	11.4 ± 6.4	8.0 ± 9.9	14.8 ± 11.0	0.058
	**z_landmarks [mm]**	0.9 ± 5.9	-3.5 ± 5.9	1.3 ± 6.1	-1.6 ± 4.1	1.8 ± 5.0	0.103
Computed 3D Measure (Central Bone Axes)	**xz-angle [°]**	-3.0 ± 24.1	-5.6 ± 16.0	-12.8 ± 17.3	-6.7 ± 28.3	-26.1 ± 14.2	0.043
	**yz-angle [°]**	-6.2 ± 11.8	-0.7 ± 7.2	-11.5 ± 8.4	-7.9 ± 5.0	-8.3 ± 7.1	0.015
	**xy-angle [°]**	-5.4 ± 23.5	4.0 ± 33.6	-9.4 ± 21.6	-6.3 ± 32.2	-8.7 ± 27.4	0.703
Manual 2D Measures	**LMD [mm]**	-3.2 ± 2.4	-0.1 ± 2.2	-4.0 ± 3.1	-1.4 ± 2.7	-4.3 ± 2.8	**< 0.001 [4]**
	**LTP/LFC [mm]**	-3.0 ± 4.7	-0.9 ± 3.5	-6.8 ± 3.8	-2.4 ± 3.5	-7.7 ± 4.3	**< 0.001 [5]**
	**MMD [mm]**	-1.9 ± 2.7	-0.6 ± 2.4	-3.3 ± 2.3	-1.9 ± 1.4	-3.9 ± 2.1	**< 0.001 [6]**
	**MTP/MFC [mm]**	-1.8 ± 3.3	-0.9 ± 4.2	-3.6 ± 3.0	-2.6 ± 2.2	-5.8 ± 2.9	**< 0.001 [7]**

Supplementary Table S3: Post-hoc details of absolute differences of computed 3D and manual 2D measures. Following repeated measures ANOVA, Tukey's multiple comparisons test was performed to detail statistical significances between absolute differences of each measure versus the ACL-intact δ_0 configuration. Accordingly, the absolute differences (Δ_x) of the respective joint configurations were compared in a pair-wise manner. Numbers in square brackets indicate the measure given in **Supplementary Table S2**. Level of significance was set to p≤0.01 and further stratified into 0.01≤p<0.001 (**) and p≤0.001 (***).

**Tukey's post-hoc test**	**Computed 3D Measures**			**Manual 2D Measures**			
	**length_ABI [1]**	**y_ABI [2]**	**length_landmarks [3]**	**LMD [4]**	**LTP/LFC [5]**	**MMD [6]**	**MTP/MFC [7]**
**Δ_{1int} vs $\Delta_{0partACL}$**	ns	ns	ns	*	ns	ns	ns
**Δ_{1int} vs $\Delta_{1partACL}$**	ns	ns	ns	ns	ns	ns	ns
**Δ_{1int} vs $\Delta_{1compACL}$**	ns	ns	ns	ns	ns	ns	ns
**Δ_{1int} vs $\Delta_{0compACL}$**	ns	***	ns	ns	*	*	**
**$\Delta_{0partACL}$ vs $\Delta_{1partACL}$**	ns	ns	**	**	**	**	ns
**$\Delta_{0partACL}$ vs $\Delta_{0compACL}$**	ns	ns	ns	ns	ns	ns	ns
**$\Delta_{0partACL}$ vs $\Delta_{1compACL}$**	ns	***	***	**	***	***	***
**$\Delta_{1partACL}$ vs $\Delta_{0compACL}$**	ns	ns	ns	ns	*	ns	ns
**$\Delta_{1partACL}$ vs $\Delta_{1compACL}$**	ns	ns	ns	ns	ns	ns	ns
**$\Delta_{0compACL}$ vs $\Delta_{1compACL}$**	ns	***	ns	ns	**	*	*

Supplementary Table S4: Absolute differences between loaded and unloaded configurations as a function of ACL condition. For any measure, the absolute difference (Δ_x) of the loaded configuration (δ_1) was referenced to the unloaded configuration (δ_0) within the respective ACL condition, i.e., ACL-intact, partially or completely ACL-deficient. Δ_x was calculated as Δ_x[ACL condition]=δ_1[ACL condition]-δ_0[ACL condition]. Data are means ± standard deviations. Repeated measures ANOVA was used to test for statistical significance between the absolute differences and no significant differences were found. Note that the manual 2D measurements by the two readers were pooled.
Please refer to **Table 2** for an explanation of the abbreviations.

Category of Measure	Measure [Unit]	absolute differences Δ_x			
		**intact**	**partial ACL deficiency**	**complete ACL deficiency**	**p-value**
Computed 3D Measure (Axis-Bone-Intersections)	**length_ABI [mm]**	0.8 ± 5.0	5.9 ± 3.9	2.2 ± 5.2	0.088
	**x_ABI [mm]**	0.6 ± 7.0	-1.9 ± 7.6	3.0 ± 9.8	0.483
	**y_ABI [mm]**	2.0 ± 9.1	6.4 ± 8.2	10.2 ± 6.8	0.085
	**z_ABI [mm]**	2.4 ± 6.1	-4.9 ± 2.8	-3.0 ± 5.3	0.438
Computed 3D Measure (Anatomic Landmarks)	**length_landmarks [mm]**	2.2 ± 4.3	6.6 ± 3.4	5.6 ± 3.5	0.030
	**x_landmarks [mm]**	0.2 ± 5.1	0.2 ± 5.4	-2.3 ± 6.2	0.388
	**y_landmarks [mm]**	3.3 ± 11.2	4.4 ± 12.1	6.8 ± 12.4	0.738
	**z_landmarks [mm]**	0.9 ± 5.9	4.8 ± 5.4	4.0 ± 6.0	0.277
Computed 3D Measure (Central Bone Axes)	**xz-angle [°]**	-3.0 ± 24.1	-7.2 ± 15.1	-19.5 ± 19.7	0.239
	**yz-angle [°]**	-6.2 ± 11.8	-10.8 ± 8.7	-0.4 ± 5.0	0.014
	**xy-angle [°]**	-5.4 ± 23.5	-13.4 ± 33.3	-2.4 ± 32.7	0.723
Manual 2D Measures	**LMD [mm]**	-3.3 ± 2.4	-3.9 ± 4.3	-2.9 ± 2.3	0.757
	**LTP/LFC [mm]**	-3.0 ± 4.7	-5.9 ± 5.0	-5.3 ± 4.0	0.345
	**MMD [mm]**	-1.9 ± 2.7	-2.7 ± 1.6	-2.0 ± 1.8	0.554
	**MTP/MFC [mm]**	-1.8 ± 3.3	-2.6 ± 3.0	-3.2 ± 2.2	0.512
